# Neurological, metabolic and inflammatory phenotypes in a mouse model of ECHS1 deficiency

**DOI:** 10.1093/braincomms/fcaf487

**Published:** 2025-12-12

**Authors:** Meghan M Eller, Aamir R Zuberi, Xiaorong Fu, Alina P Montalbano, Felix Nitschke, Shawn C Burgess, Cat Lutz, Rachel M Bailey

**Affiliations:** Graduate School of Biomedical Sciences, University of Texas Southwestern Medical Center, Dallas, TX 75235, USA; Center for Alzheimer’s and Neurodegenerative Diseases, University of Texas Southwestern Medical Center, Dallas, TX 75235, USA; The Jackson Laboratory Center for Precision Genetics, The Jackson Laboratory, Bar Harbor, ME 04609, USA; Center for Human Nutrition, University of Texas Southwestern Medical Center, Dallas, TX 75235, USA; Department of Pediatrics, University of Texas Southwestern Medical Center, Dallas, TX 75235, USA; Department of Pediatrics, University of Texas Southwestern Medical Center, Dallas, TX 75235, USA; Department of Biochemistry, University of Texas Southwestern Medical Center, Dallas, TX 75235, USA; Center for Human Nutrition, University of Texas Southwestern Medical Center, Dallas, TX 75235, USA; Department of Pharmacology, University of Texas Southwestern Medical Center, Dallas, TX 75235, USA; The Jackson Laboratory Center for Precision Genetics, The Jackson Laboratory, Bar Harbor, ME 04609, USA; Center for Alzheimer’s and Neurodegenerative Diseases, University of Texas Southwestern Medical Center, Dallas, TX 75235, USA; Department of Pediatrics, University of Texas Southwestern Medical Center, Dallas, TX 75235, USA

**Keywords:** ECHS1, epilepsy, EEG, metabolism, inflammation

## Abstract

ECHS1 deficiency (ECHS1D) is a rare and devastating neurometabolic disease that currently has no defined treatments. This disorder results from missense loss-of-function mutations in the *ECHS1* gene that results in severe developmental delays, encephalopathy, hypotonia and early death. ECHS1 enzymatic activity is necessary for the beta-oxidation of fatty acids and the oxidation of branched-chain amino acids within the inner mitochondrial matrix. The pathogenesis of disease remains poorly understood. To expand our knowledge on disease mechanisms, we generated a novel mouse model of ECHS1D that possesses a disease-associated variant knocked-in (KI) the *Echs1* allele and a knock-out (KO) of the other *Echs1* allele. Neurological and metabolic abnormalities were assessed under basal conditions, and acute inflammation was tested as a potential disease driver. Mice containing KI/KI or KI/KO alleles were viable with normal development and survival, and the combined KI and KO alleles resulted in more than a 95% reduction of Echs1 protein levels. ECHS1D mice had significantly increased epileptiform EEG activity and were sensitive to seizure induction, which resulted in the death of 60% of ECHS1D mice. Power spectral analysis revealed ECHS1D mice had increased slow-wave EEG power that was associated with sleep dysfunction. Under basal conditions, energy status and mitochondrial function within the brain was unaffected, while aromatic amino acid content was increased. Markers of neuroinflammation were increased in ECHS1D mice in an age-dependent manner and acute inflammatory challenge resulted in failure to thrive and early lethality in ECHS1D mice. In conclusion, we developed a novel model of ECHS1D that can be used to study disease mechanisms and for therapeutic development.

## Teaser

Eller *et al*. report the generation of a novel mouse model of ECHS1 deficiency that recapitulates features of human disease, including sleep disturbances, epileptic activity and inflammation sensitivity. They use this model to uncover metabolic and inflammatory abnormalities that may underlie disease progression.

## Graphical Abstract

**Graphical Abstract:**
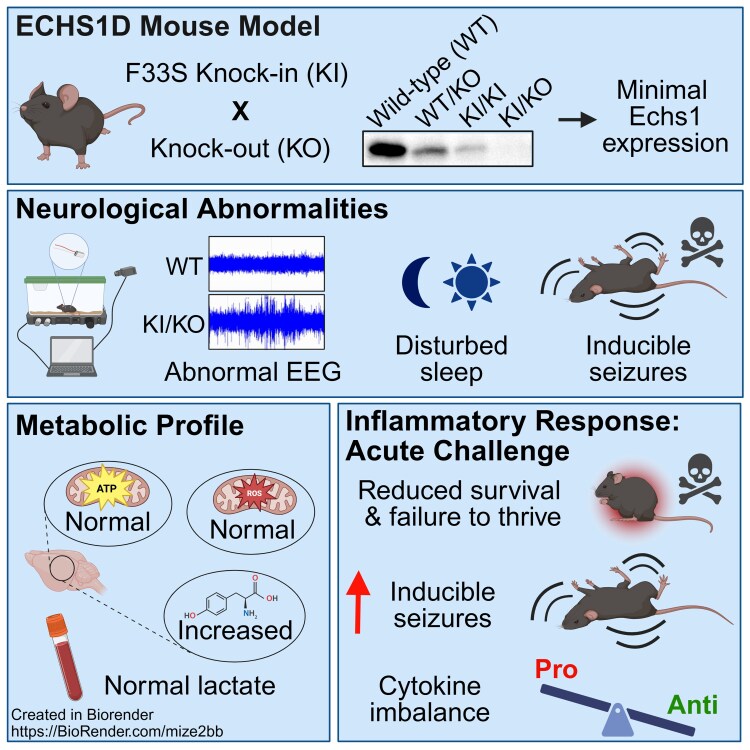

## Introduction

ECHS1 deficiency (ECHS1D) is a rare, autosomal recessive disorder caused by loss-of-function mutations in *ECHS1*. Disease-associated variants influence various factors such as splice sites, mitochondrial localization, protein folding and stability or substrate binding. Since its discovery in 2014, over 100 pathogenic or likely pathogenic variants have been identified.^1^  *ECHS1* is a nuclear DNA gene encoding enoyl-coenzyme A (CoA) hydratase, Short chain 1 or ECHS1, which translocates to the inner mitochondrial matrix and assembles as a homohexamer. ECHS1 has a confirmed role in the beta-oxidation of fatty acids and breakdown of valine, as well as a proposed role in leucine and isoleucine catabolism.^2^

ECHS1D has a Leigh-syndrome-like presentation including severe developmental delays or regressions, hypotonia, dystonia, severe infantile-onset epilepsy and failure to thrive.^3,4^ Due to the heterogeneity of this disorder, it is divided into four main phenotypes ranging from most to least severe: perinatal early fatal, severe infantile, slowly progressive infantile and exercise-induced dystonia.^5^ Each type is distinguished by the age of onset and severity of progression. The perinatal form is the most commonly reported, with onset shortly after birth and rapid fatality, whereas the infantile subtypes present within the first few months of life with variable rates of disease progression. Lesions within the basal ganglia, primarily globus pallidus and caudate nucleus, are detected in most patients irrespective of phenotype classification.^1^ Elevated brain lactate and cortical and cerebellar degeneration are primarily limited to the perinatal subtype.^5,6^ Few ECHS1D patients have undergone post-mortem assessment, limiting our knowledge on ECHS1D specific neuropathology, but macroscopic necrosis and microscopic vacuolization have been reported.^6,7^

Dysregulated mitochondrial function is a hallmark of numerous metabolic disorders and can contribute to disease pathogenesis. In disorders of general branched-chain amino acid (BCAA) metabolism such as maple syrup urine disease, BCAA concentrations are elevated, resulting in an overall amino acid imbalance that has downstream impacts on mitochondrial health.^8^ This contrasts with ECHS1D, in which patients exhibit accumulation of valine-derived metabolites, while leucine and isoleucine metabolism appear to be unaffected.^2,9^ Elevation of toxic cysteine/cysteamine conjugates from valine intermediates is hypothesized to directly interfere with mitochondrial function through inhibition of pyruvate dehydrogenase complex (PDC).^10,11^ Assessments of patient-derived fibroblasts have demonstrated reduced activity of the PDC and oxidative phosphorylation (OXPHOS) complexes, although the extent of these deficiencies varies among individuals.^6,10,12^ Additional studies have shown that ECHS1 knock-out (KO) cells have reduced OXPHOS complex assembly and mitochondrial oxygen consumption, implicating mitochondrial respiratory defects in ECHS1D pathogenesis.^13^

Oxidative stress resulting from mitochondrial damage can trigger aberrant inflammatory signalling, while inflammation itself can further exacerbate mitochondrial damage.^14-16^ In patients with inborn errors of metabolism, infectious stressors frequently precipitate metabolic decompensation due to heightened energy demands.^17^ Consistent with this, ECHS1D patients often report symptom onset or worsening following illness, implicating inflammatory responses as potential modulators of disease progression.^5,18^ Supporting this concept, inhibition of inflammatory signalling in animal models of Leigh syndrome attenuates disease severity, suggesting inflammation may contribute to pathogenesis.^19^  ^,20^ However, a direct link between inflammatory responses and clinical manifestations in ECHS1D has not yet been established.^21^

In this study, we uncovered potential mechanisms contributing to disease progression using a novel animal model of ECHS1D. ECHS1D mice had disrupted EEG activity and sleep architecture, increased seizure susceptibility and mild neuroinflammation. Interestingly, under basal conditions, brain energy status and mitochondrial function remained intact. Challenge with an acute inflammatory agent exacerbated the epileptic phenotype and induced death, providing evidence for inflammation as a modulator of disease progression. These findings detail novel phenotypes of ECHS1D in mice and identify potential mechanisms of disease, the knowledge of which can guide therapeutic development.

## Materials and methods

### Experimental animals

All procedures were performed in accordance with protocols approved by the Institutional Animal Care and Use Committee of the University of Texas Southwestern Medical Center (UTSW) and the Jackson Laboratory, both AAALAC-accredited facilities. Targeted mutations in the Echs1 gene were generated at the Jackson Laboratory on a C57BL/6J background using Clustered Regularly Interspaced Short Palindromic Repeats/CRISPR-associated protein 9 (CRISPR/Cas9) genome editing. Single-cell zygotes were electroporated with Cas9 protein, single guide RNA and a mutagenic donor oligonucleotide (Supplemental Table 1). Founder mice were generated, and genome editing was scored after sequencing the targeted locus. Founders containing the F33S knocked-in (KI) allele were mated to C57BL/6J mice to confirm the germ line transmission in N1 progeny, and the resulting heterozygous KI mice were backcrossed once more to C57BL/6J. The viability of homozygous mice was evaluated by intercrossing N2 heterozygous mice. Two Echs1 mutant strains were recovered and assigned with Jackson Laboratory Stock numbers. Stock 35254 (C57BL/6J-Echs1em1Lutzy/Mmjax) contains the A31A and F33S edited alleles, and Stock 35256 (C57BL/6J-Echs1em3Lutzy/Mmjax) contains a single indel in Exon 2 that is predicted to generate a frameshift null allele. Strain 35254 was homozygous viable with both male and female mice fertile. Strain 35256 was homozygous lethal (0 homozygous mice observed from 65 progeny generated from the intercross of heterozygous males and females). Breeders of both strains were transferred to UTSW and maintained under controlled environmental conditions, 12-h light-dark cycles and provided food and water *ad libitum*. Wild-type (WT) littermates were used as controls and order of testing or sample collection was randomized among genotypes for all experiments.

### Echs1 expression

At 2 months of age, mice were deeply anaesthetized with tribromoethanol and perfused with phosphate buffered saline (PBS) with heparin. Tissues were immediately frozen on dry ice and stored at −80°C. Protein was extracted and analysed via western blotting. Blots were incubated with mouse anti-ECHS1 (1:1000; Proteintech, #66117-1-IG) followed by mouse peroxidase secondary antibody (1:10 000; Jackson Immuno #115-035-146). Protein loading was normalized to actin (primary 1:10 000, Cell Signaling #4970S; secondary 1:10 000 Jackson Immuno #111-035-144) or GAPDH (primary 1:10 000, Meridian #H68504M; secondary 1:10 000 Jackson Immuno #115-035-146). Quantification was performed using ImageJ (version 2.1.0).

### Telemetry recording

Mice were implanted with HD-X02 telemetry probes (Data Systems International) using intended coordinates LH: AP +1.0, ML −1.5, RH: AP −2.0, ML +2.0. After at least 1 week of recovery, dural recordings were acquired over a 24-h period consisting of one dark and one light cycle. Data were acquired using Ponemah and analysed in Neuroscore using the same settings across groups. Sleep scoring was performed using the Rodent Sleep Scoring 2 programme and total power was determined by the software. Epileptic spikes and spike trains were quantified using the Spike Analysis programme.

### Pentylenetetrazol seizure induction

The pentylenetetrazol (PTZ) kindling model was performed as described.^22^ Briefly, mice received an intraperitoneal (i.p.) injection of 30 mg/kg PTZ dissolved in sterile saline every other day for a total of 12 injections. Mice were observed for 30 min following injection and seizure severity was scored using a standard Racine scale. Evaluators were blinded to the genotype of mice for all testing.

### Focused microwave brain fixation and harvest

At 3 months of age, mice were euthanized using a 5 kW Muromachi Microwave Fixation System (MMW-05, Muromachi Kikai Co., Ltd., Tokyo, Japan) to preserve the metabolic profile by rapidly inactivate enzymes involved in the metabolism of metabolites and neurotransmitters.^23,24^ Briefly, the unanaesthetized mouse was restrained in an animal holder where the head was positioned in a water-jacketed cone (WJM-28, Muromachi Kikai Co., Ltd.) and the body immobilized using a plunger. The animal holder was carefully positioned in the applicator head of the MMW-05 unit and a high-energy microwave (5 kW) was used to irradiate the head of the mouse for 1.40 s. The microwave-fixed brain was immediately excised and frozen in liquid nitrogen.

### Measurement of nucleotides and short-chain acyl-CoAs

Ion-pairing reverse-phase liquid chromatography (LC) ionization-tandem mass spectrometry was used to quantify adenine nucleotides and short-chain acyl-CoAs as previously reported.^25^ Frozen brain samples were spiked with stable-isotope-labelled ATP, adenosine monophosphate, acetyl-CoA and malonyl-CoA internal standards and homogenized in 0.4 M HClO_4_ (perchloric acid) containing 0.5 mM ethylene glycol tetraacetic acid. Neutralized supernatants were subjected to LC-MS/MS (liquid chromatography-tandem mass spectrometry) analysis using a Shimadzu LC-20AD LC system coupled to a Triple Quad™ 5500+ QTrap LC-MS/MS (Applied Biosystems/Sciex Instruments). A reverse-phase C18 column (Waters Atlantis T3, 150 × 2.1 mm, 3 mm) was used with an LC mobile phase consisting of water/methanol (95:5, v/v) with 4 mM DBAA (Eluent A) and water/acetonitrile (25:75, v/v; Eluent B). Multiple-reaction monitoring in positive ion mode was used to detect each analyte and its stable isotope internal standard.

### Measurement of amino acids

Frozen brain samples were spiked with stable-isotope labelled amino acid internal standards (Isotec) and quantified using LC-MS/MS as we previously reported.^26^ Tissue was homogenized in 4% perchloric acid, and supernatant was neutralized with 0.5 M K_2_CO_3_. After salt removal, supernatants were derivatized using established protocols.^27^ Amino acids were separated on a reverse-phase C18 column (Waters, Xbridge, 150 × 2.1 mm, 3.0 μm) with gradient elution and detected using the multiple-reaction monitoring mode by monitoring specific transitions under positive electrospray on a Triple Quad™ 5500+ QTrap LC-MS/MS (Applied Biosystems/Sciex Instruments). Amino acids were quantified by comparing individual ion peak areas to that of the stable isotope internal standard.

### Measurement of mitochondrial respiration

At 3 months of age, mice were euthanized via cervical dislocation, mitochondria were isolated from fresh brain tissue and oxygen-consumption rates were measured using Seahorse XF96 Analyzer as described.^28^ Five micrograms of mitochondria were loaded per well.

### Magnetic resonance spectroscopy

At 9 months of age, T2 FLAIR images were obtained using ^1^H frequency (300 MHz) on the Agilent (Varian) 7.0 T MR imaging system. A 3 mm × 3 mm × 3 mm voxel was placed over the midbrain for spectroscopy measures and was consistent between animals. All metabolite concentrations were normalized to water.

### Neuroinflammation quantification

For transcriptional analysis, RNA was extracted using the RNeasy Plus Mini Kit according to manufacturer’s instructions (Qiagen, #74136). RNA was DNase treated **(**NEB, #M0303) and stored at −80°C or immediately used for cDNA synthesis (Roche, #04896866001). cDNA was quantified by quantitative polymerase chain reaction (qPCR) using PowerUp SYBR Green Master Mix (ThermoFisher, #A25741) on an Applied Biosciences StepOne Plus qPCR Thermocycler. Custom primers were used to quantify Iba1 and glial fibrillary acidic protein (GFAP). Actin was quantified using PrimePCR Assay Mmu ActB (Biorad, #10025636).

Iba1 Fwd: 5′—AGCAGGGATTTGCAGGGAGG—3′

Iba1 Rev: 5′—TCCATGTACTTCACCTTGAAGGC—3′

GFAP Fwd: 5′—CTGAGGCAGAAGCTCCAAGATG—3′

GFAP Rev: 5′—CGGAGTTCTCGAACTTCCTCCTC—3′

### Histology

At 12 months of age, mice were deeply anaesthetized with tribromoethanol and perfused with PBS-heparin. Whole brain was drop-fixed in 10% neutral buffered formalin for 48 h and then processed, paraffin embedded and microtome sectioned at 5-µm thickness. Immunohistochemical (IHC) staining was performed using standard techniques. Tris antigen masking solution (Vector Laboratories, #H-3301) was used for antigen retrieval. Primary antibody used was anti-Iba1 (1:1000; Fujifilm, #1919741) and secondary antibody was biotinylated goat anti-rabbit secondary antibody (1:1000; Vector Laboratories, BA-1000). Staining was visualized with a Vecta stain ABC HRP Kit (Vector Laboratories, PK-6100) and DAB kit (Sigma, #D4418). Counter staining with haematoxylin was performed prior to cover slipping with poly-mount xylene (Fisher Scientific, #NC0825496).

### Imaging and analysis

Light microscopy images were captured at 20× on a Hamamatsu Nanozoomer 2.0 HT. Microglia activation status was determined using the microglial activation algorithm within HALO (Halo2.2. Indica Labs, Albuquerque, NM, USA). The midbrain and globus pallidus were outlined for analysis and the same outline and threshold settings were applied to each image. Evaluators were blinded to genotype for analysis.

### Lipopolysaccharide challenge

Lipopolysaccharide (LPS) from *Escherichia coli* O55:B5 (Sigma, L2880) was dissolved in sterile PBS at 0.5 mg/ml. At 2 months of age, baseline weights were recorded, and mice were randomly assigned to receive an i.p. injection of sterile saline or 8 mg/kg LPS. Weights were collected daily for the first week following injection, and once a week thereafter.

### Cytokine quantification

Blood was collected via facial vein 4 h after saline or LPS treatment and serum was isolated. A custom Bio-Plex Pro Mouse Cytokine Express Assay was used to quantify cytokine concentrations.

### Statistical analysis

Power analyses were performed to determine sample size. One-way or two-way ANOVA with Tukey or Sidak’s multiple comparison was used for multiple group comparisons and Geisser–Greenhouse’s epsilon correction was used if the estimated epsilon was < 0.75. Student’s *t*-test was used for comparisons between two groups with equal variance, and Welch’s *t*-test was used for comparisons between two groups with significantly different variance. Binomial test was used to compare the observed distribution between groups in two categories. Survival differences were analysed using the log rank test. Statistical significance was set at *P* ≤ 0.05. Outliers were determined using Grubbs’ test and excluded. Values are expressed as mean ± standard error of the mean (SEM).

## Results

### Development of the ECHS1D mouse model

#### Generation of ECHS1D mice

**Figure 1:**
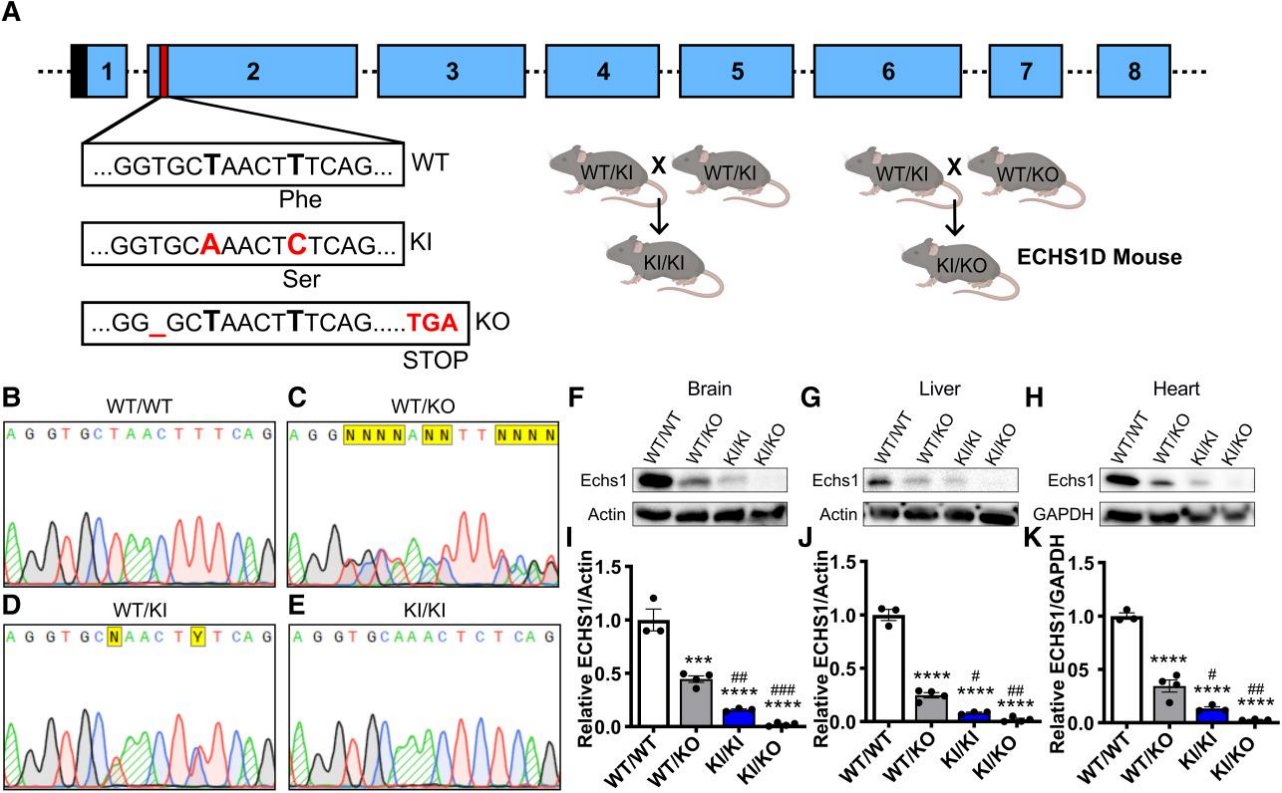
**Generation and validation of ECHS1D mouse model.** ECHS1D mice were generated from Echs1 KI and Echs1 KO lines. (**A**) Depiction of mouse Echs1 gene, with Exons 1–8. Black = mitochondrial localization signal; red = target region. Compared with the WT allele, the F33S KI allele has a synonymous mutation (c.93T > A; p.A31A) and a missense mutation (c.98T > C; p.F33S). The KO allele has a frameshift mutation (c.90delT; p.G30del). Heterozygous KI mice (WT/KI) were bred to establish homozygous KI mice (KI/KI). WT/KI mice were also bred with heterozygous KO (WT/KO) mice to generate the KI/KO line. Created in BioRender. Bailey (2025) https://BioRender.com/xoo20za (**B–E**). Sequencing of genomic DNA showing the presence of the mutations within transgenic mice. Striped traces = A; N = base cannot be determined; Y = base is either C or T. ECHS1 expression in tissues was assessed in 2-month-old mice (*N* = 3–4/genotype). (**F–H**) Total protein from brain (**F**; *N* = 3 WT, 4 WT/KO, 3 KI/KI, 4 KI/KO), liver (**G**; *N* = 3 WT, 4 WT/KO, 3 KI/KI, 4 KI/KO) and heart (**H**; *N* = 3 WT, 4 WT/KO, 3 KI/KI, 3 KI/KO) was analysed via western blot. (**I–K**) Band intensity was quantified and ECHS1 was normalized to actin or GAPDH and set relative to WT levels. Blots were cropped for clarity and full images are provided in Supplemental Fig. 2. Each dot represents an individual mouse with SEM indicated. One-way ANOVA with Tukey’s *post hoc* analysis, ***P < 0.001, ****P < 0.0001 compared with WT/WT; ^#^*P* < 0.05, ^##^*P* < 0.01, ^###^*P* < 0.001 compared with WT/KO.

There are currently no published rodent models that genetically mimic ECHS1D as complete Echs1 KO is embryonic lethal. While heterozygous KO mice possess a 50% reduction in Echs1 expression, they develop normally with only mild lipid accumulation and a cardiomyopathy phenotype.^29,30^ To establish a model of disease, CRISPR/Cas9 was used to generate a KI mouse line that contains a disease-associated variant (c.98T > C; p.F33S) (Fig. 1).^6^ The F33S variant was selected because it is conserved between mice and humans and has been identified in a compound heterozygous patient that exhibits infant onset Leigh syndrome symptoms and a significant reduction in Echs1 protein levels without neonatal mortality.^6^ Further, due to its position at the start of Exon 2, we simultaneously generated a KO line that possesses a frameshift mutation to result in an early stop codon that prevents the translation of functional protein. Heterozygous KI mice were bred together to generate homozygous KI mice, or with heterozygous KO mice to establish the compound heterozygous ECHS1D mouse as depicted in Fig. 1. While we were unable to obtain homozygous KO mice due to embryonic lethality, KI/KI and KI/KO mice were viable and fertile. Sanger sequencing was used to confirm germline mutations in mice (Fig. 1B–E). ECHS1D mice developed normally, without an outward phenotype and had similar body weights as WT littermates (Supplemental Fig. 1).

#### Model validation

In ECHS1D patients, genetic variants typically result in the loss of ECHS1 expression and subsequent function.^6,12^ To determine if the introduced mutations affect Echs1 expression in our model, protein was quantified in brain, liver and heart (Fig. 1). The KI mutation significantly decreased Echs1 protein in a gene-dose dependent fashion (Fig. 1F–H, Supplemental Fig. 2). Across tissues, WT/KO mice retained 50% of WT Echs1 as expected, while KI/KI mice expressed ∼12% and KI/KO mice had 3% of WT levels. As KI/KI mice were largely phenotypically normal and with higher Echs1 expression, subsequent assessments focused on KI/KO mice, now referred to as ECHS1D mice. This analysis supports that the F33S mutation results in a significant decrease in Echs1 protein levels and when combined with the KO allele results in a considerable loss of functional Echs1 throughout the body.

### Epileptic phenotype in ECHS1D mice

#### EEG activity

**Figure 2:**
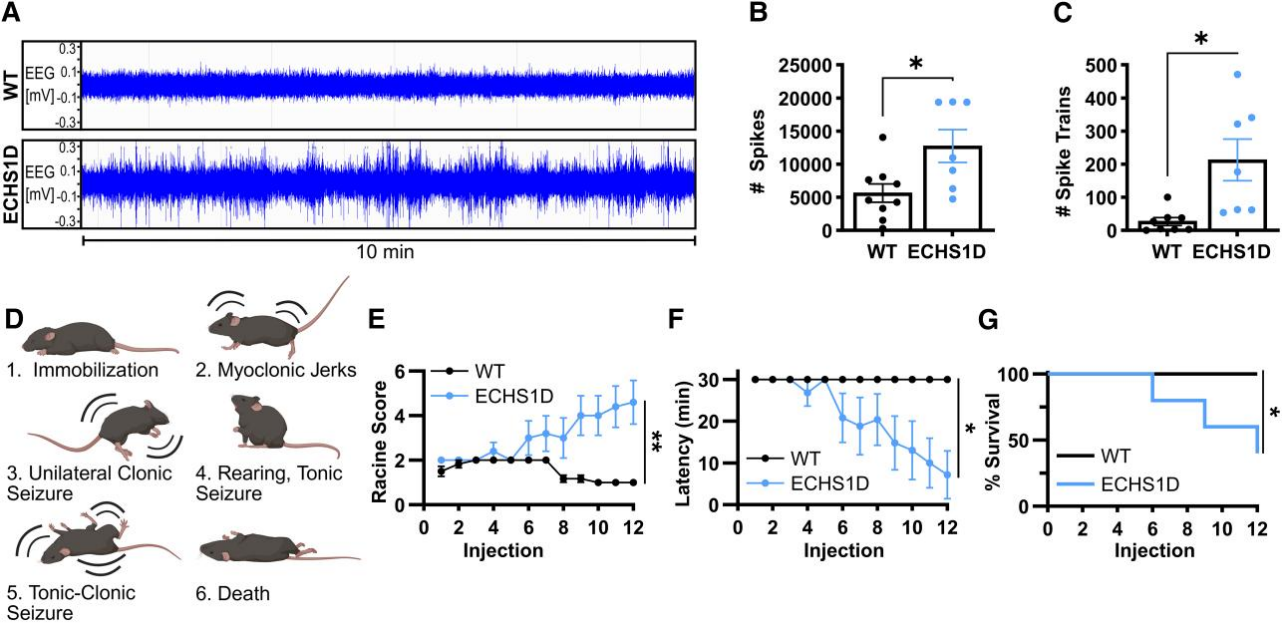
**ECHS1D mice have abnormal baseline EEG.** At ∼3.5 months of age, mice received wireless telemetry implants and EEG activity was recorded over a 24-h period [*N* = 9 WT (4F, 5M), 7 ECHS1D (3F, 4M)]. (**A**) Representative raw EEG traces from WT and ECHS1D. (**B, C**) Individual spikes (**B**) and spike trains (**C**) were quantified. Each dot represents an individual mouse with SEM indicated. (**D**) Depiction of Racine scale used to score seizure severity following PTZ seizure induction. Created in BioRender. Bailey (2025) https://BioRender.com/z5g1jzp (**E–G**) Mice received 12 injections of PTZ (30 mg/kg) every other day and seizure severity (**E**), latency (**F**) and survival (**G**) was recorded following each injection [*N* = 6 WT (3F, 3M), 5 ECHS1D (4F, 1M)]. (**B**) Student’s *t*-test, *P < 0.05 (**C**) Welch’s *t*-test, *P < 0.05; (**E–G**) Two-way ANOVA, Genotype effect *P < 0.05, **P < 0.01. (**D**) Log rank test, *P < 0.05.

EEG recordings of Leigh syndrome patients show disorganized background neural activity,^31^ and ∼40% of ECHS1D patients develop epilepsy throughout their disease progression.^3^ To assess brain activity of ECHS1D mice, we performed EEG and EMG recordings for 24 h using wireless telemetry implants. Representative raw EEG traces shown in Fig. 2A demonstrate that ECHS1D mice have significantly increased, abnormal EEG signal compared with WT mice. Quantification of epileptic spikes and spike trains confirmed a significant increase in epileptiform discharges in ECHS1D mice when compared with WT (Fig. 2B and C).

#### Seizure susceptibility

Spontaneous seizures were not observed in ECHS1D mice; however, the altered baseline EEG suggests ECHS1D mice may be more susceptible to seizure onset. To test this, we performed a kindling induction paradigm using PTZ.^22^ Seizure severity was scored using the Racine scale depicted in Fig. 2D. With the low dose used (30 mg/kg), WT mice failed to develop seizures. In contrast, ECHS1D mice developed progressive seizures that worsened over time with a significant reduction in latency to seize (Fig. 2E and F). Severe seizures in ECHS1D were associated with early lethality where 60% of mice died by injection 12, while WT mice had 100% survival (Fig. 2G). These results demonstrate that under standard conditions ECHS1D mice have increased epileptiform activity that is associated with increased seizure susceptibility following PTZ treatment.

### Sleep dysfunction in ECHS1D mice

**Figure 3:**
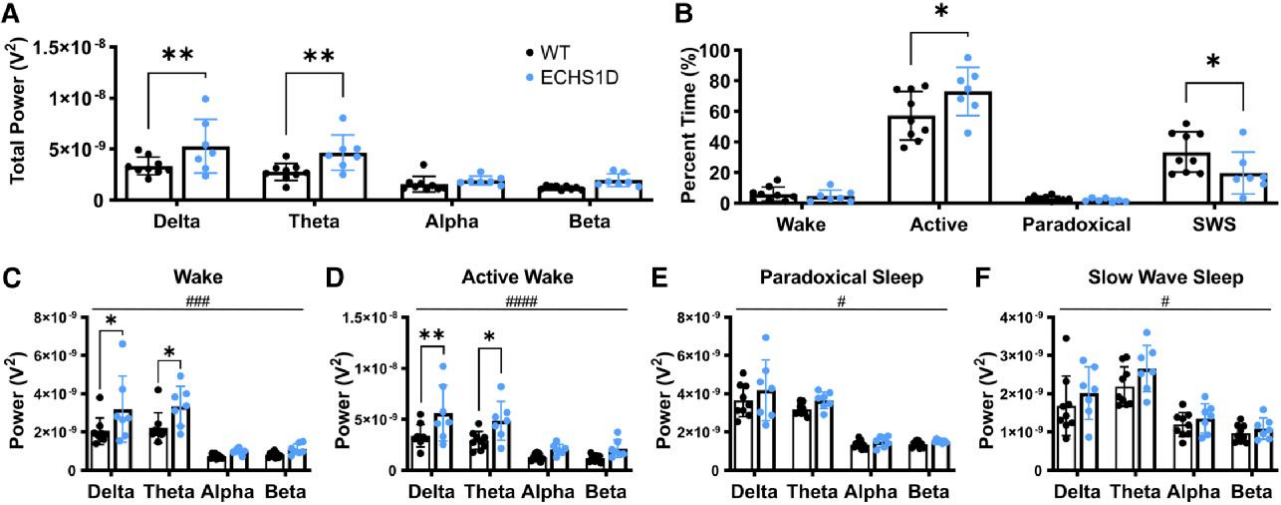
**ECHS1D mice have increased slow-wave power and disrupted sleep.** At ∼3.5 months of age, mice received wireless telemetry implants and EEG activity was recorded over a 24-h period [*N* = 9 WT (4F, 5M), 7 ECHS1D (3F, 4M)]. (**A**) Total EEG power from delta, theta, alpha, or beta frequencies. (**B**) Sleep staging was performed and per cent time spent in wake, active wake, paradoxical sleep, or slow-wave sleep was calculated. (**C–F**) Total EEG power from delta, theta, alpha, or beta frequencies was calculated within each sleep stage. Each dot represents an individual mouse with SEM indicated. Two-way ANOVA with Sidak’s multiple comparisons, genotype effect, ^#^*P* < 0.05, ^###^*P* < 0.001, ^####^*P* < 0.0001, WT versus ECHS1D, *P < 0.05, **P < 0.01.

In addition to spike activity, generalized slowing is the most common EEG abnormality in epileptic patients.^32^ To determine if this was present in ECHS1D mice, power analysis was performed. Overall, ECHS1D mice had significantly elevated delta and theta power compared with WT mice (Fig. 3A).^4,33,34^ To determine if these power alterations result in sleep disruption in our model, the percentage time spent in each sleep stage was calculated. ECHS1D mice spent significantly more time in the active awake stage, where animals are awake and moving, and similar amounts of time in the wake stage, where animals are awake but not active, when compared with WT mice. Conversely, ECHS1D mice had a significantly reduced percentage of slow-wave sleep compared with WT mice, although paradoxical sleep was similar (Fig. 3B). When power analysis was performed within sleep stages, ECHS1D mice were significantly different from WT mice in all stages with slow-wave power (delta and theta waves) being significantly increased during wake and active wake (Fig. 3C–F). Together these results support that ECHS1D mice have abnormal brain activity characterized by increased slow-wave power, which is associated with sleep dysfunction.

### Neurometabolic abnormalities in ECHS1D mice

#### Brain lactate levels

**Figure 4:**
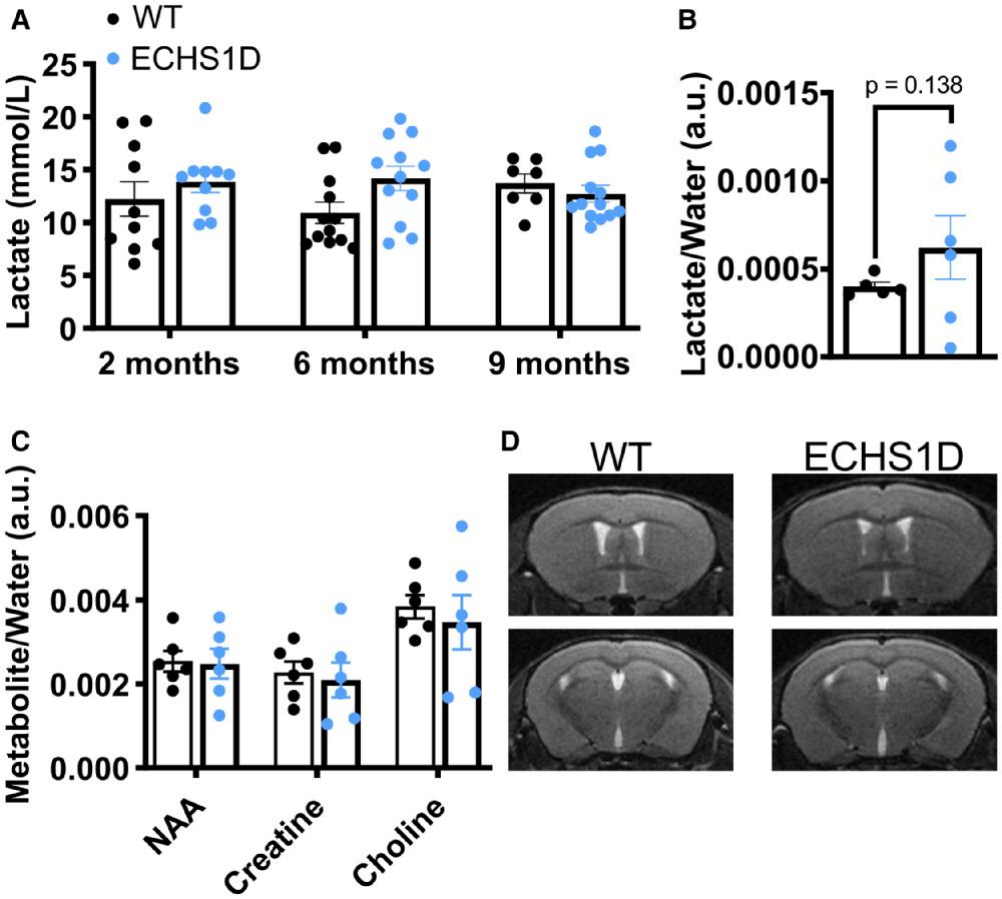
**Serum and brain lactate levels in ECHS1D mice.** (**A**) Lactate was quantified from serum isolated at 2, 6 and 9 months of age from WT or ECHS1D mice [2 months, *N* = 10 WT (5F, 5M), 10 ECHS1D (5F, 5M); 6 months, *N* = 12 WT (5F, 7M), 12 KI/KO (6F, 6M); 9 months *N* = 7 WT (3F, 4M), 13 ECHS1D (8F, 5M)]. (**B–D**) At 9-months of age, WT and ECHS1D mice were subjected to magnetic resonance spectroscopy quantification of brain lactate (**B**), NAA, creatine and choline (**C**) [*N* = 5 WT (2F, 3M), 6 ECHS1D (3F, 3M)]. Metabolites were normalized to the signal from water. Each dot represents an individual mouse with SEM indicated. (**D**) Representative T2-weighted FLAIR MRI images. Top = representative rostral section of striatal regions, Bottom = midline section of hippocampal and thalamic regions. (**A, C**) Two-way ANOVA with Sidak’s multiple comparisons and (**B**) Welch’s *t*-test, *P* = 0.138.

ECHS1D patients often present with elevated circulating lactate^6,35,36^ and brain lactate.^10^ To determine if this is recapitulated in our model, blood lactate was quantified in WT and ECHS1D mice across aging (Fig. 4A). There were no differences in circulating lactate between groups; however, blood lactate does not reliably inform on brain lactate.^37^ To quantify brain lactate, we performed magnetic resonance spectroscopy on 9-month-old mice. While brain lactate levels were increased in the majority of ECHS1D mice when compared with WT mice, some ECHS1D mice had lower than normal brain lactate levels (Fig. 4B). We also found that concentrations of N-acetylaspartate (NAA), creatine and choline were similar between groups, and that the brain appeared structurally normal in ECHS1D mice (Fig. 4C and D). Together these data suggest that lactic acidosis likely does not significantly contribute to the baseline ECHS1D mouse phenotype.

#### Energy metabolism

**Figure 5:**
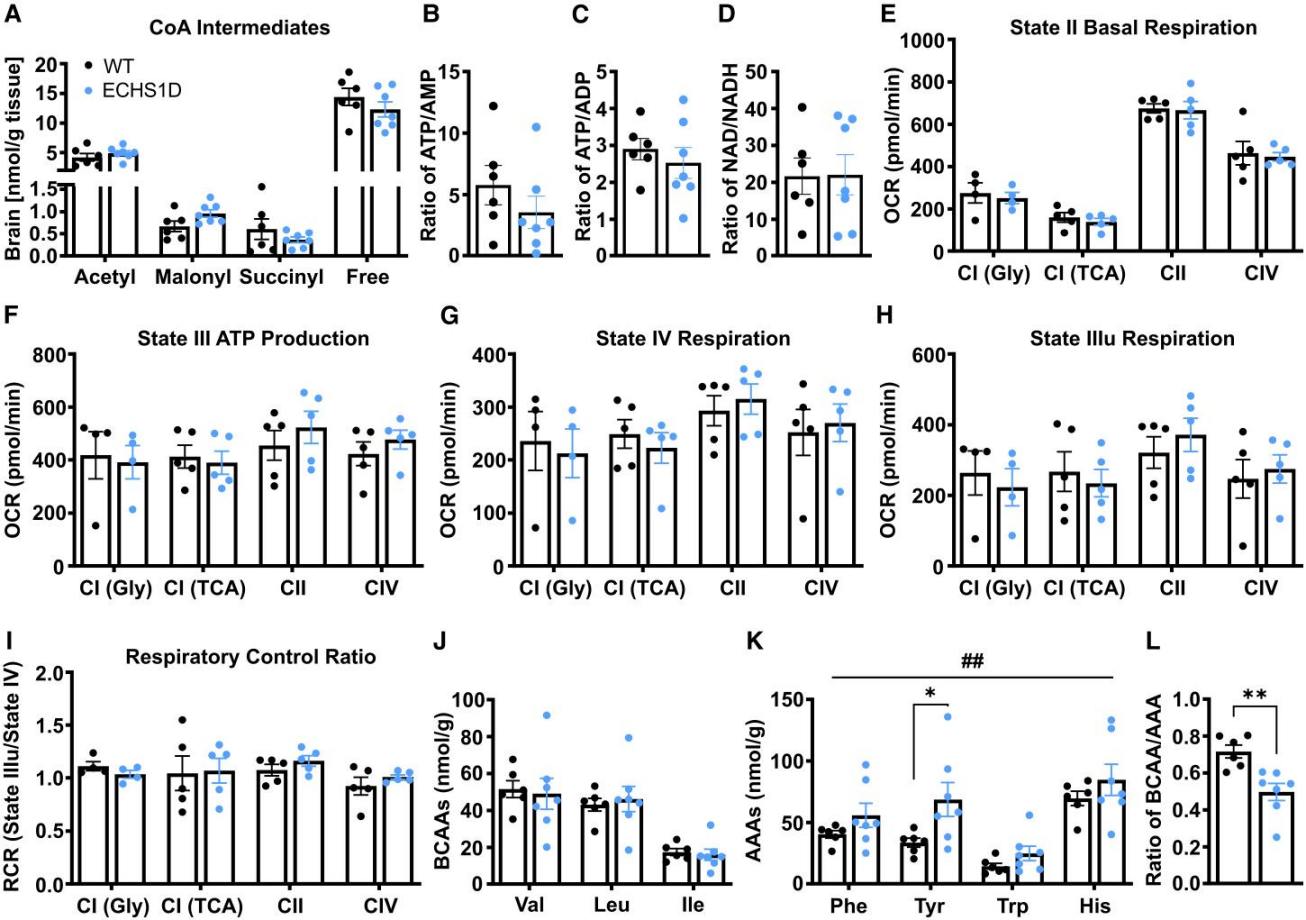
**Brain energy metabolism is unaffected in ECHS1D mice, while AAA content is increased.** Following microwave-assisted fixation, brain samples from 3-month-old WT and ECHS1D mice [*N* = 6 WT (3F, 3M), 7 ECHS1D (4F, 3M)] were analysed via LC-MS/MS for quantification of energy charge and amino acids. (**A–D**) Concentrations of CoA metabolites, energy charge and redox status in brain tissues. (**E–I**) Mitochondria were isolated from WT and ECHS1D brains collected at ∼4 months of age, and respiration assessed using a Seahorse XF96 Analyzer [*N* = 5 WT (3F, 2M), 5 ECHS1D (3F, 2M)]; 5 µg of mitochondria were plated with substrates specific for each complex: CI (Gly) = pyruvate and malate; CI (TCA) = glutamate/malate; CII = succinate/rotenone; CIV = ascorbate/tetramethylphenylenediamine/antimycin A. The final respiration rate following injection of antimycin and rotenone was subtracted from each measurement. (**E**) Baseline oxygen-consumption rate (OCR) in the presence of substrate without adenosine diphosphate (ADP). (**F**) Maximal OCR following addition of ADP. (**G**) Maximal OCR after uncoupling with CCCP treatment. (**H**) OCR after ATP synthase inhibition with oligomycin. (**I**) Respiratory control ratio was calculated by dividing State III uncoupled OCR to State IV OCR, representing coupling efficiency. (**J**) Total concentration of individual BCAAs in 3-month-old microwave-fixed brain samples. (**K**) Total concentration of individual AAAs. (**L**) Total concentration of BCAAs (valine, isoleucine and leucine) was divided by total concentration of AAAs (phenylalanine, tryptophan, histidine and tyrosine) to obtain BCAA/AAA ratio. Each dot represents an individual mouse with SEM indicated. (**K**) Two-way ANOVA with Sidak's multiple comparisons, Genotype effect **^##^***P* < 0.01; WT versus ECHS1D *P < 0.05. L: Student’s *t*-test, *P < 0.05.

Energy failure caused by mitochondrial dysfunction is a prominent cause of epilepsy across mitochondrial disorders, and epileptic activity can further induce energy depletion.^38,39^ Additionally, as a nutrient-sensing enzyme,^40^ changes in ECHS1 activity can cause metabolic dysregulation.^41,42^ To assess brain metabolism under basal conditions in ECHS1D mice, energy metabolites and amino acids were measured in tissues from 3-month-old mice (Fig. 5).^23-25^ Acyl-CoA metabolites, energy charge and redox status were unchanged between WT and ECHS1D mice, demonstrating the maintenance of normal energy levels in this model (Fig. 5A–D). Steady-state levels of ATP, however, do not necessarily inform metabolic shifts in OXPHOS rates.^43^ To directly assess energy production in ECHS1D mice, we isolated mitochondria from WT and ECHS1D brain tissue and performed a Seahorse extracellular flux assay. There were no differences in any respiration parameters though, indicating preserved mitochondrial function in ECHS1D mice under basal conditions (Fig. 5E–I).

#### Amino acid metabolism

Due to the role of ECHS1 in BCAA catabolism, we additionally measured brain amino acid content following microwave-assisted fixation. While most amino acid concentrations, including BCAAs and their respective proportions, were unchanged, ECHS1D mice had an overall increase of aromatic amino acid (AAA) content with a significant elevation of tyrosine (Fig. 5J and K, Supplemental Figs 3 and 4). Consequently, the ratio of BCAAs to AAAs was significantly reduced (Fig. 5G). As ECHS1D patient samples commonly have secondary deficiency of PDC activity,^12,44^ we calculated the alanine and proline ratios that are often used as biomarkers in PDC deficiencies.^45,46^ We found that the alanine:leucine/lysine ratios were increased in the brains of ECHS1D mice, while the proline ratios were unchanged (Supplemental Fig. 4). The diagnostic cut-off for PDC deficiency is based on measurements obtained from blood samples;^45,46^ it remains unclear how the ratios within the brain are impacted by PDC dysfunction.

Taken together, these data suggest that under basal conditions, brain energy production and amino acid metabolism is largely unaffected in ECHS1D mice. However, reduction in BCAA/AAA ratio is associated with systemic inflammation, suggesting that inflammatory signalling may be altered in this model.^47^

### Neuroinflammation in ECHS1D mice

**Figure 6:**
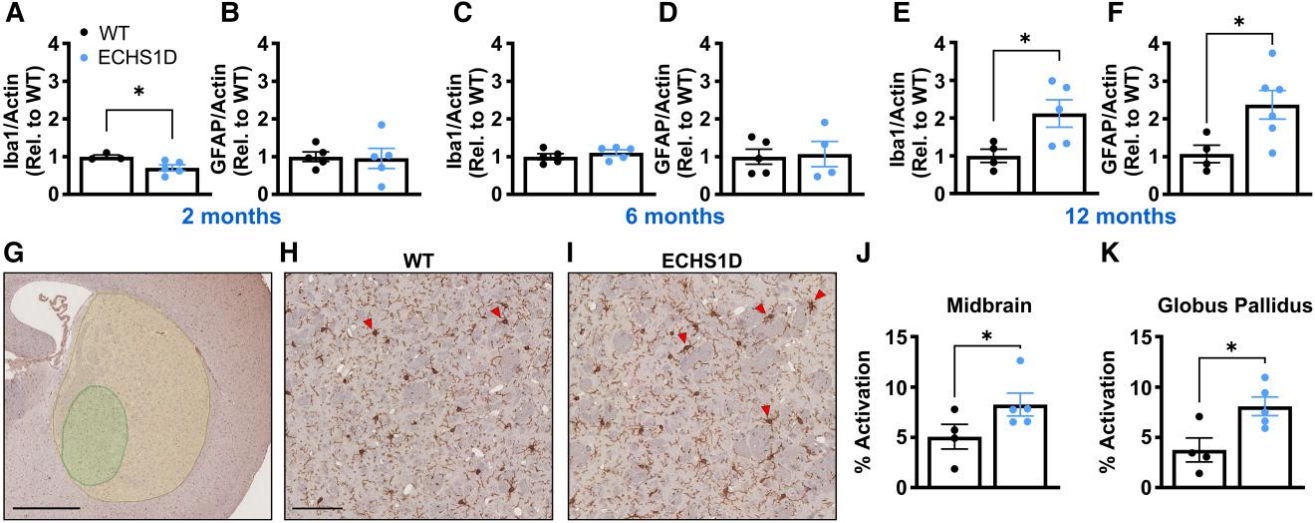
**Neuroinflammation markers are increased in aged ECHS1D mice.** (**A–F**) Midbrain was collected from WT and ECHS1D mice (*N* = 3–5/genotype) at 2 (**A**, **B**), 6 (**C**, **D**), or 12 (**E**, **F**) months of age and cDNA synthesized from total RNA was analysed via qPCR. Expression of Iba1 (**A**, **C**, **E**) and GFAP (**B**, **D**, **F**) was normalized to actin and set relative to WT levels. Each dot represents an individual mouse with SEM indicated. (**G–K**) IbaI staining was performed on sections from 12-month-old mice to visualize microglia. (**G**) Representative image of areas used for analysis. Large circle is midbrain region and small circle is globus pallidus. Scale bar = 1 mm. (**H**, **I**) Representative images of activated microglia, characterized by larger cell bodies and bushy protrusions. Red arrowheads point to activated microglia. Scale bar = 100 µm. (**J**, **K**) Per cent of activated microglia in midbrain (**J**) and globus pallidus (**K**). Student’s *t*-test, *P < 0.05. (**A)**  *N* = 3 WT (2F, 1M), 5 ECHS1D (4F, 1M); (**B**) *N* = 5 WT (3F, 2M), 5 ECHS1D (4F, 1M); (**C**) *N* = 5 WT (3F, 2M), 5 ECHS1D (1F, 4M); (**D**) *N* = 5 WT (3F, 2M), 4 ECHS1D (1F, 3M); (**E**) 4 WT (4F), 5 ECHS1D (3F, 2M); (**F**) 4 WT (4F), 6 ECHS1D (4F, 2M); J-K: 4 WT (2F, 2M), 5 ECHS1D (3F, 2M).

Neuroinflammation is hypothesized to contribute to disease progression in Leigh syndrome and Leigh-syndrome-like disorders,^19,48^ but this has not been directly tested for ECHS1D. To determine if neuroinflammation is increased in ECHS1D mice, mRNA expression of Iba1 and GFAP was measured in the midbrain. Interestingly, at 2 months of age, Iba1 expression was significantly reduced in ECHS1D mice compared with WT, while GFAP expression was unaffected (Fig. 6A and B). Given the progressive nature of ECHS1D in patients,^5,6^ we analysed additional samples collected at 6 and 12 months of age. Both inflammatory markers were similar between WT and ECHS1D mice at 6 months of age (Fig. 6C and D), while expression of Iba1 and GFAP was approximately doubled in ECHS1D mice at 12 months of age (Fig. 6E and F).

To further assess the neuroinflammation in our model, IHC staining was performed against Iba1 in a separate cohort of 12-month-old mice (Fig. 6G–K). Activation of microglia was assessed in the midbrain to remain consistent with the bulk region used for qPCR analysis and specifically in the globus pallidus as this region is affected in ECHS1D.^1^ Consistent with increased Iba1 transcripts, ECHS1D mice had a significant increase in percentage of activated microglia when compared with WT (Fig. 6H–K). This effect was similar between the midbrain and globus pallidus. These data suggests that under basal conditions there is a slow progression of neuroinflammation in ECHS1D mice, marked by increased inflammatory markers and microglial activation upon aging.

### Inflammation sensitivity

**Figure 7:**
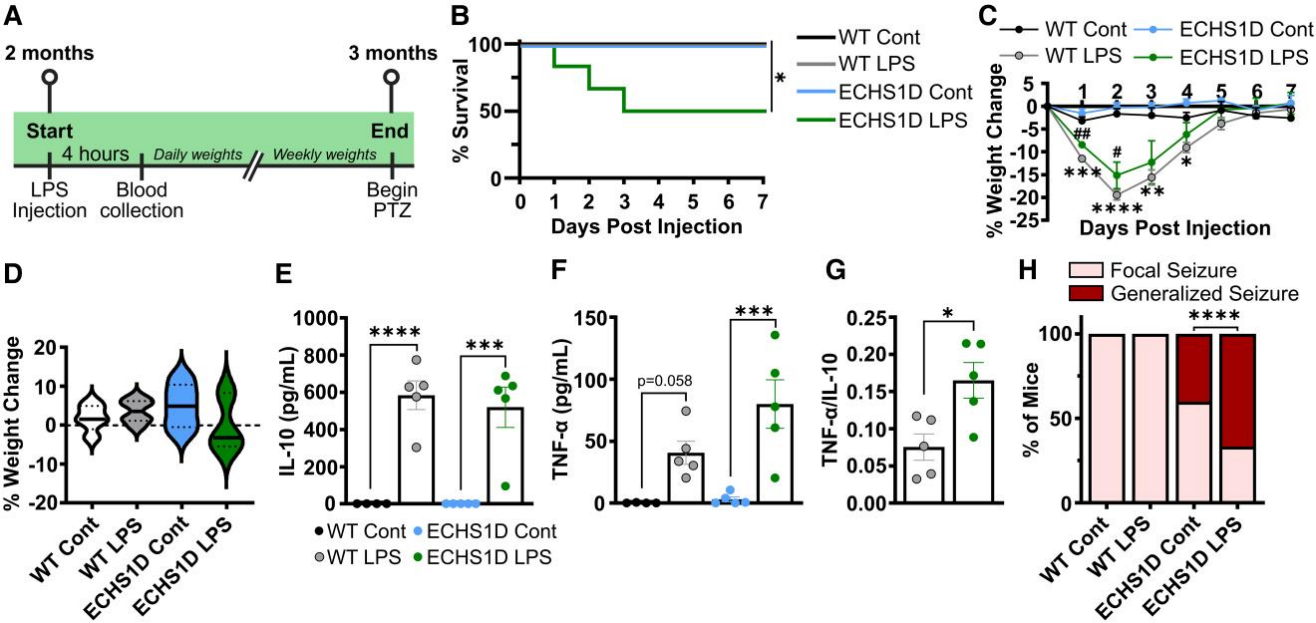
**ECHS1D mice have reduced survival, inflammation imbalance and increased seizure susceptibility following LPS administration.** At 2 months of age, WT and ECHS1D mice received an intraperitoneal injection of LPS [8 mg/kg; *N* = 5 WT (2F, 3M), 6 ECHS1D (4F, 2M)] or saline Cont; [*N* = 5 WT (2F, 3M), 5 ECHS1D (3F, 2M)] (**A**) Study design. Created in BioRender. Bailey (2025) https://BioRender.com/l7qfi1v (**B**) Per cent survival following LPS treatment. Survival rates were stable past Day 7 and before PTZ treatment. (**C**) Per cent change in body weight in the first week following injection. (**D**) Per cent change from baseline body weight in surviving mice at 1-month post-injection [*N* = 5 WT Cont, 5 ECHS1D Cont, 6 WT LPS, 3 ECHS1D LPS (2F, 1M)]. (**E–G**) At 4 h post-treatment, blood was collected via facial vein and serum cytokines were quantified. (**G**) Ratio of TNF-α to IL-10. Each dot represents an individual mouse with SEM indicated. (**H**) Percentage of surviving mice that had focal seizures (Score 0–2) or generalized seizures (Score 3–6) on the final PTZ injection. (**B**) Log rank test, *P < 0.05; (**C**) Two-way ANOVA with Tukey’s multiple comparisons, WT Cont. versus WT 8 mg/kg *P < 0.05, **P < 0.01, ***P < 0.001, ****P < 0.0001, ECHS1D Cont. versus ECHS1D 8 mg/kg, ^#^*P* < 0.05, ^##^*P* < 0.01; (**E**, **F**) Two-way ANOVA with Sidak's multiple comparisons, Cont versus LPS ***P < 0.001, ****P < 0.0001, (**G**) Student's *t*-test, *P < 0.05; (**H**) Binomial test, ****P < 0.0001.

The reduced BCAA/AAA ratio in young adult mice and subsequent age-dependent neuroinflammation suggests that ECHS1D mice may inherently have increased sensitivity to inflammatory stress. This phenomenon has been observed in Leigh syndrome and ECHS1D patients, in which symptoms worsen following infection.^3,49^ To assess if inflammation worsens symptoms of ECHS1D mice, animals were given a single i.p. injection of LPS. LPS causes an acute inflammatory response by triggering the release of inflammatory cytokines in various cell types and results in long-lasting neuroinflammation.^50^ Mice were monitored for weight loss and survival for 1-month following injection and then subjected to PTZ seizure induction (Fig. 7A). While LPS treatment was well-tolerated in control mice with 100% survival, ECHS1D mice had poorer outcomes in which survival was reduced to 50% within 3 days following injection (Fig. 7B). Compared with ECHS1D saline-treated mice, LPS-treated mice remained lower in body weight in the weeks following treatment, indicating a failure to thrive. This was not observed in WT controls who gained weight at a similar rate regardless of treatment (Fig. 7C and D). Movement abnormalities were not observed in any mice in the 1-month following LPS treatment.

To determine if altered immune responses underlie this sensitivity, inflammatory cytokines were quantified in serum samples obtained 4 h after LPS treatment. There were no cytokines detected in saline-treated mice, demonstrating that under basal conditions, inflammatory signalling is typical in ECHS1D mice. Following LPS, cytokine levels were increased in both WT and ECHS1D mice as expected (Fig. 7E and F). The concentration of the anti-inflammatory cytokine interleukin-10 (IL-10) was unchanged between groups, while there was a trending increase in the pro-inflammatory cytokine tumor necrosis factor-alpha (TNF-α). The ratio of pro- to anti-inflammatory cytokines is often used as an indicator of immune regulation.^51^ Interestingly, ECHS1D mice have an elevated TNF-α/IL-10 ratio compared with WT (Fig. 7G), suggesting that the inflammatory balance is shifted towards a pro-inflammatory state in this model following a stressor such as LPS.

To determine if inflammation exacerbates neurological abnormalities in ECHS1D mice, the surviving mice underwent the seizure induction paradigm at 1-month post-treatment. Although saline and LPS-treated ECHS1D mice had similar seizure severity at the start of the paradigm, by the last injection 66% of ECHS1D LPS-treated mice exhibited generalized seizures (racine score above 3), compared with only 40% of control ECHS1D mice. Importantly, LPS did not alter the seizure susceptibility of WT controls (Fig. 7H). These data demonstrate that like patients, ECHS1D mice are hypersensitive to an acute inflammatory response that results in early lethality, failure to thrive and worsening of symptoms.

## Discussion

This study is the first to report a genetically relevant mouse model of ECHS1D, a rare Leigh-syndrome-like disorder. Due to the lethality of *Echs1* KO,^29,30^ the effects of Echs1 reduction have only been minimally investigated in heterozygous KO mice. A 50% reduction in Echs1 causes cardiomyopathy phenotypes and lipid accumulation within liver and kidney.^29,30^ Cardiac impairments are not observed in all patient reports and are mostly limited to severe neonatal cases.^3,6,10,36^ Herein, we focused on assessing neurological phenotypes as ECHS1D patients present with Leigh-like symptoms; however, future studies warrant investigation of the cardiac and lipid accumulation phenotypes that have been reported in other ECHS1D models.^29,30^

The presence of the F33S knock-in variant significantly decreased Echs1 protein levels which was compounded by the KO allele. To date, one patient with infant onset ECHS1D has been reported to be a compound heterozygote that possesses the F33S variant on one allele and the N59S variant on the other allele.^6^ ECHS1 expression was significantly reduced in that patient’s fibroblasts, similar to what we observed in our mice, although it is unknown how the N59S variant impacted ECHS1 expression. This patient presented with epileptic seizures within the first few days of life followed by developmental delays and hypotonia and was reported to be alive at 3 years of age in 2015.^6^ Similar to this patient, epilepsy was a prominent phenotype in the ECHS1D mouse model, marked by significantly increased epileptiform discharges and severe seizures following low dose administration of the chemoconvulsant, PTZ. The shared epileptic activity between the patient and ECHS1D mice supports the clinical relevance of this phenotype in this model.

Atypical EEG activity is common among mitochondrial disorders, with generalized slowing marked by increased low frequency power as the primary manifestation.^31,32,52^ ECHS1D mice had significantly elevated delta and theta power, specifically during periods of wake. Delta and theta power typically increase with extended wakefulness and decrease during sleep.^33,53^ In line with this, ECHS1D mice spent significantly more time in the active wake period and reduced time in slow-wave sleep. This suggests ECHS1D mice may have sleep disturbances that result in increased slow-wave power during wake periods. Disrupted sleep has been reported in literature in one ECHS1D patient to date,^4^ and this has been further supported through personal communication with patients’ families who note impaired sleep as a feature of their child’s disease. While sleep disturbances are common among mitochondrial disease patients,^54^ the cause of this symptom in ECHS1D remains to be determined.^54,55^ Interestingly, when Echs1 is selectively knocked down in glia, it results in sleep loss in flies.^56^ This further supports that sleep disturbances may be directly linked to the loss of ECHS1 expression, with recent work from flies suggesting a role for glial ECHS1 activity.

Given that ECHS1 loss impairs mitochondrial energy production in cells,^13^ we hypothesized that ATP depletion within the brain may underlie the abnormal brain activity. Utilization of a fixation approach to preserve the metabolic profile in frozen brain tissue^23^ revealed that ATP levels are well-maintained in ECHS1D mice under basal conditions. This was further supported by demonstrating intact mitochondrial respiration. Together, these results suggest that mitochondrial dysfunction does not drive the abnormal brain activity in this model. These findings are in line with reports from more mildly affected patients, who often display normal respiration profiles.^57^ Currently, respiration deficits appear to be limited to KO cell models and fibroblasts from more severe patients.^13^ It should be noted that the respiration measures in our studies were performed using isolated mitochondria rather than intact primary cells, which allowed us to directly assess mitochondrial function without the influence of other cellular components.^58^ Future studies should investigate ECHS1D mouse primary neurons to determine the effect of non-mitochondrial components in respiration defects as well as if mitochondrial dysfunction arises following disease stressors.

Severely affected ECHS1D patients often have secondary deficiency of PDC,^12,44^ which is associated with high lactate levels and elevated circulating alanine and proline.^45,46,59^ Given the other phenotypes in this model that suggest it aligns with infantile-onset patients, we did not expect PDC activity to be impacted. In support of this, blood lactate levels were normal. Additionally, the activity of Complex I in the presence of pyruvate is dependent on proper acetyl-CoA formation by the PDC.^60^ As Complex I activity was preserved in ECHS1D brain mitochondria, this further supports that PDC activity is maintained under baseline conditions.^60^ Although a subset of ECHS1D mice had elevated brain lactate and alanine:leucine/lysine ratios were increased, any potential PDC deficiency is likely very mild given our other findings. Additional studies are needed to confirm this.

Individual brain amino acid concentrations were largely unchanged, but the BCAA/AAA ratio was significantly reduced due to elevated AAA content.^61-63^ Increased AAA content within the brain has been linked to systemic inflammation.^47^ Under basal conditions, young adult ECHS1D mice did not have elevated neuroinflammation or circulating cytokines compared with WT, suggesting that in the absence of stressors, inflammatory signalling is typical in ECHS1D mice. GFAP and Iba1 transcripts were significantly increased by 1 year of age, which was associated with a significant increase in microglial activation in ECHS1D mice as assessed by Iba1 staining. Aging is associated with disrupted mitochondrial function and increased neuroinflammation in healthy controls.^64-66^ The increase in neuroinflammatory markers in older ECHS1D mice suggests that infantile-onset ECHS1D patients may be more sensitive to age-related declines in brain health.

Compared with patients with severe neonatal onset, the outward phenotype of ECHS1D mice was relatively mild under basal conditions and more similar to infantile or later-onset patients. This provides the opportunity to address disease mechanisms and therapeutic interventions that can either prevent or halt progression of the neurological phenotypes. A common feature of mitochondrial diseases is metabolic decompensation following an energy intensive trigger, which may uncover or exacerbate disease symptoms. In line with this, many infantile or later-onset ECHS1D patients do not report symptoms until an immunological insult, such as a vaccination or infection.^5,18^ The mild phenotype of ECHS1D mice provided the opportunity to directly test if acute inflammation can trigger new symptoms or worsen the existing phenotype.^5,18^ To test if our model responds like these infantile-onset patients, mice were treated with LPS. Similar to patients, ECHS1D mice displayed sensitivity to inflammatory insults, shown by reduced survival, failure to thrive and increased seizure susceptibility following LPS treatment. Furthermore, the increased ratio of pro- to anti-inflammatory cytokines compared with WT mice suggests loss of Echs1 results in imbalanced immune responses which may account for the poorer outcomes following infection. Additional studies that assess inflammatory responses following the rescue of Echs1 expression are necessary to demonstrate causality between inflammatory stress and the observed outcomes in this model.

The phenotype of the ECHS1D mouse model reported herein was mild and representative of the infantile-onset patients, despite an almost complete reduction in Echs1 expression. This model enables evaluation of disease progression and therapeutic development in both infantile-onset and milder forms of ECHS1D, which represent the longest-lived patient populations. Due to the typical survival, weight gain and gross development in ECHS1D mice, we focused our initial characterization of mice to 2–3 months of age. This was driven, in part, by the use of wireless telemetry devices to measure EEG and EMG activity. These devices cannot be implanted in mice under 20 g,^67^ which prohibits the assessment of younger animals with this approach. This is a limitation of the current work and future studies will assess brain development and disease-driving mechanisms in neonatal and adolescent mice. Another limitation is that F33S KI/KO ECHS1D mice do not model the pathogenesis of the more frequently occurring neonatal onset subtype. As complete Echs1 KO is embryonic lethal, a more severe mouse model could be generated via knock-in of variants associated with neonatal onset or by inducible KO during a postnatal period.

## Conclusion

We developed a novel model of ECHS1D that displays infantile-onset patient-relevant phenotypes and identified novel ones that may be relevant to human disease. We demonstrated the use of ECHS1D mice in testing disease mechanisms, which can be used in developing therapies for patients that currently have limited treatment options. These findings are directly relevant to ECHS1D and may be applicable to other mitochondrial disorders as well.

## Supplementary Material

fcaf487_Supplementary_Data

## Data Availability

Data that support the findings of this study are available from the corresponding author, upon reasonable request.
