# Supplementary material for: Neurological, metabolic and inflammatory phenotypes in a mouse model of ECHS1 deficiency
Source: Brain Commun. 2025 Dec 12;7(6):fcaf487. doi: 10.1093/braincomms/fcaf487 (PMC12715774; doi:10.1093/braincomms/fcaf487)
Supplement: fcaf487_Supplementary_Data [file fcaf487_supplementary_data.docx]

**Supplemental Table 1: Sequences for Mouse Model Generation**

| **Oligo** | **Sequence (5’ - 3’)** |
| --- | --- |
| sgRNA | TGTACTGAAAGTTAGCACCT |
| Mutagenic Donor^a^ | GCGATGTGGTCAGGTTGATGACCAAGATTAACCAACCACGGTGACTTTAATATCTTTATGATGACACTTCCCTTTGTCCTCTTGACCTAGGTGC**A**AACT**C**TCAGTA**T**ATCATCACAGAAAAGAAAGG |
| Target Amplification (Fwd) | CTTCCACAGGTAAGGCTTGG |
| Target Amplification (Rev) | CCTTATCCCCACCAGTGAGC |

^a^DNA sequence of the donor oligonucleotide was altered to prevent guide reassociation with edited genomic DNA via the incorporation of three nucleotide changes (identified in bolded font, underlined). Founder mice only contain A31A (c.93T>A) and F33S (c.98T>C) edited alleles.


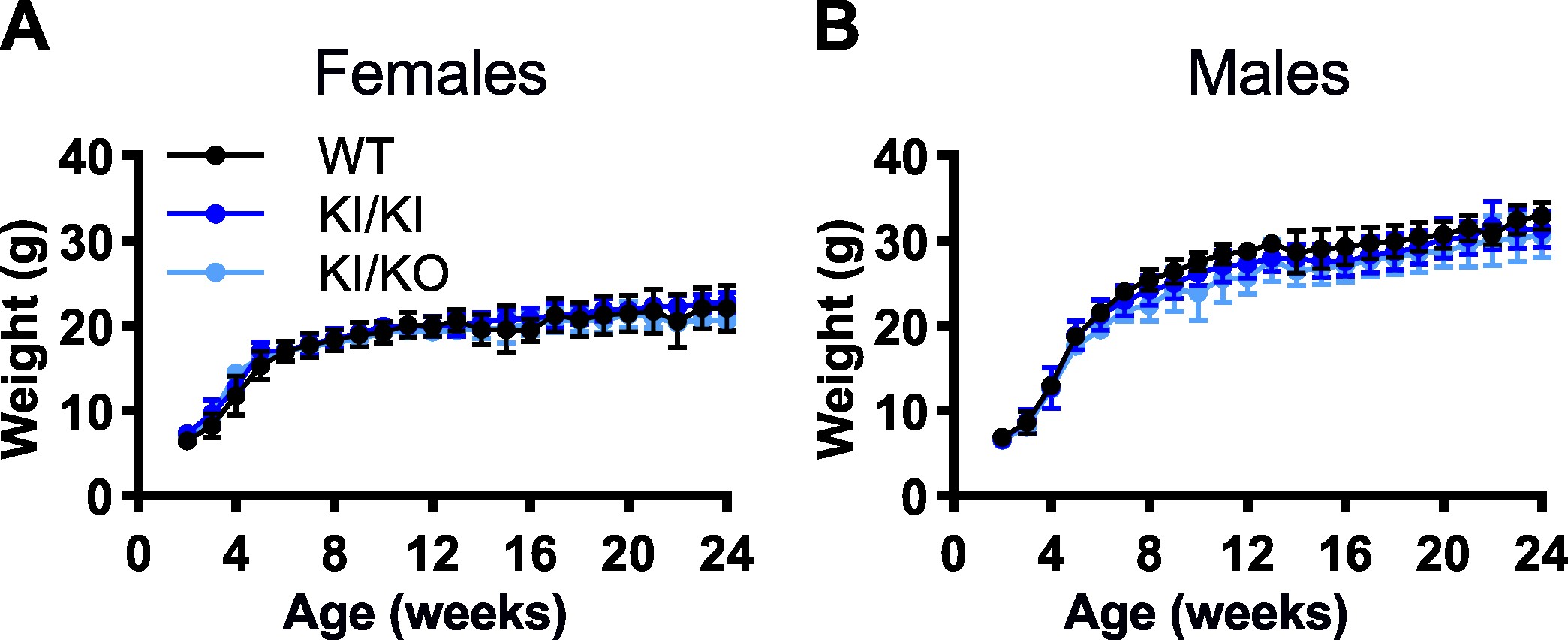


**Supplemental Figure 1: WT and ECHS1D mice have similar body weights.** Mice were weighed weekly beginning at 2 weeks of age and until at 24 weeks of age. There were no differences in weight gain or maintenance between female (A; N=10 WT, 12 KI/KI, 9 KI/KO) and male (B; N=5 WT, 8 KI/KI, 9 KI/KO) WT and ECHS1D mice. Each dot represents the average weight with SEM indicated. A: Two-way ANOVA, Genotype effect F(2,37) = 1.066, p=0.3548, B: Two-way ANOVA, Genotype effect F(2,19) = 2.247, p=0.1331


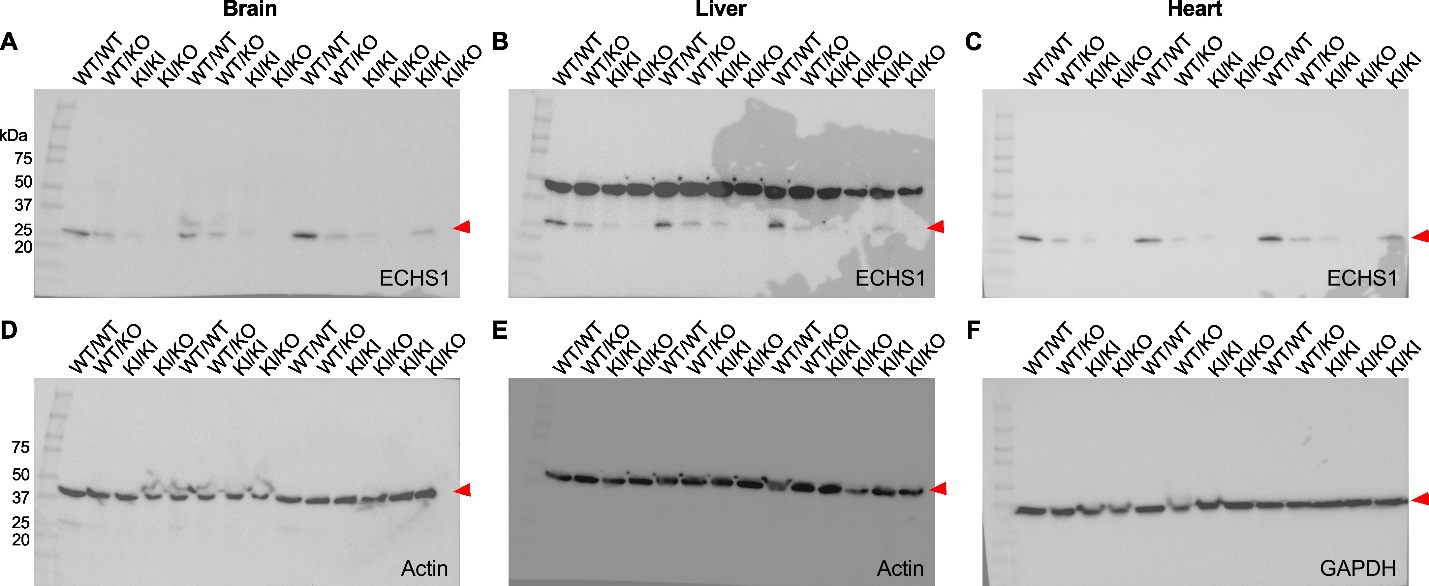


**Supplemental Figure 2: Uncropped blots used in Figure 1.** (**A-F**) Full blots that were cropped for clarity in Figure 1. Both ECHS1 and housekeeping proteins were probed on the same blot for each tissue. Red arrows indicate the band of interest.


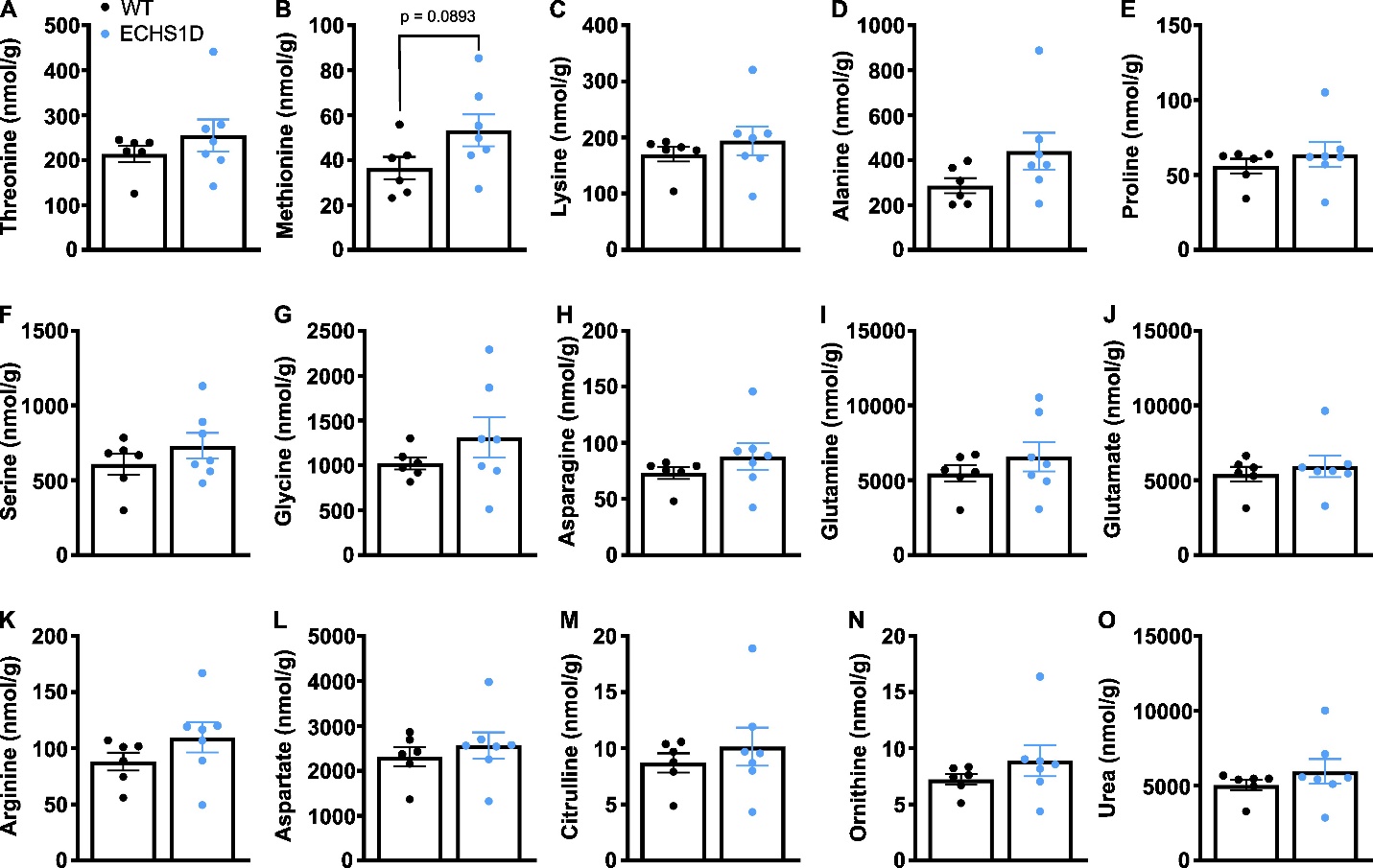


**Supplemental Figure 3: Brain amino acid content is similar between WT and ECHS1D mice**. Following microwave-assisted fixation, brain samples from 3-month-old WT and ECHS1D mice (N=6 WT[3F, 3M], 7 ECHS1D[4F, 3M]) were analyzed via GC-MS for quantification of amino acids. (**A-O**) Concentrations of individual amino acids normalized to tissue weight. Each dot represents an individual mouse with SEM indicated. Analyzed via student’s t-test.

**
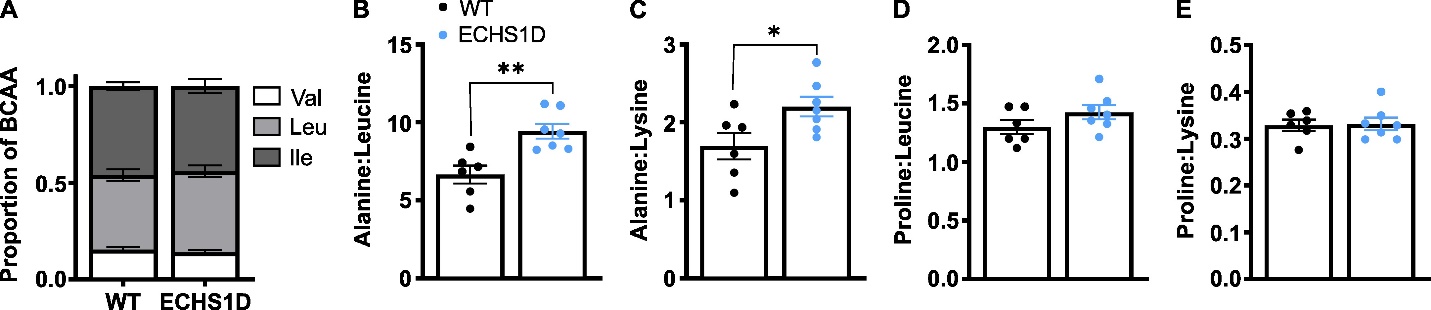
**

**Supplemental Figure 4: Amino acid ratios are mildly altered in ECHS1D mice**. Following microwave-assisted fixation, brain samples from 3-month-old WT and ECHS1D mice (N=6 WT[3F, 3M], 7 ECHS1D[4F, 3M]) were analyzed via GC-MS for quantification of amino acids. (**A**) Proportion of each BCAA. (**B-C**) Ratios of alanine to leucine (B) and lysine (C). (**D-E**) Ratios of proline to leucine (D) and lysine (E). Each dot represents an individual mouse with SEM indicated. Student's t-test, *p<0.05, **p<0.01.
